# Using three statistical methods to analyze the association between exposure to 9 compounds and obesity in children and adolescents: NHANES 2005-2010

**DOI:** 10.1186/s12940-020-00642-6

**Published:** 2020-08-31

**Authors:** Bangsheng Wu, Yi Jiang, Xiaoqing Jin, Li He

**Affiliations:** 1 Emergency Department, Zhongnan Hospital of Wuhan University, 169 Donghu Road, Wuhan, 430071 Hubei China; 2 Second Clinical School, Wuhan University, Wuhan, 430071 Hubei China; 3 Internal hematology, Zhongnan Hospital of Wuhan University, 169 Donghu Road, Wuhan, 430071 Hubei China

**Keywords:** Obesity, Adolescent, Child, Weighted quantile sum (WQS) regression, Bayesian kernel machine regression (BKMR)

## Abstract

**Background:**

Various risk factors influence obesity differently, and environmental endocrine disruption may increase the occurrence of obesity. However, most of the previous studies have considered only a unitary exposure or a set of similar exposures instead of mixed exposures, which entail complicated interactions. We utilized three statistical models to evaluate the correlations between mixed chemicals to analyze the association between 9 different chemical exposures and obesity in children and adolescents.

**Methods:**

We fitted the generalized linear regression, weighted quantile sum (WQS) regression, and Bayesian kernel machine regression (BKMR) to analyze the association between the mixed exposures and obesity in the participants aged 6–19 in the National Health and Nutrition Examination Survey (NHANES) 2005–2010.

**Results:**

In the multivariable logistic regression model, 2,5-dichlorophenol (2,5-DCP) (OR (95% CI): 1.25 (1.11, 1.40)), monoethyl phthalate (MEP) (OR (95% CI): 1.28 (1.04, 1.58)), and mono-isobutyl phthalate (MiBP) (OR (95% CI): 1.42 (1.07, 1.89)) were found to be positively associated with obesity, while methylparaben (MeP) (OR (95% CI): 0.80 (0.68, 0.94)) was negatively associated with obesity. In the multivariable linear regression, MEP was found to be positively associated with the body mass index (BMI) z-score (*β* (95% CI): 0.12 (0.02, 0.21)). In the WQS regression model, the WQS index had a significant association (OR (95% CI): 1.48 (1.16, 1.89)) with the outcome in the obesity model, in which 2,5-DCP (weighted 0.41), bisphenol A (BPA) (weighted 0.17) and MEP (weighted 0.14) all had relatively high weights. In the BKMR model, despite no statistically significant difference in the overall association between the chemical mixtures and the outcome (obesity or BMI z-score), there was nonetheless an increasing trend. 2,5-DCP and MEP were found to be positively associated with the outcome (obesity or BMI z-score), while fixing other chemicals at their median concentrations.

**Conclusion:**

Comparing the three statistical models, we found that 2,5-DCP and MEP may play an important role in obesity. Considering the advantages and disadvantages of the three statistical models, our study confirms the necessity to combine different statistical models on obesity when dealing with mixed exposures.

## Introduction

The continuous increase in obesity has become an important worldwide health problem in the past 30 years [1]. In 2016, about 18% of children and adolescents aged 5–19 were overweight or obese [2]. Obesity in children increases the risk of health conditions, such as coronary heart disease, diabetes mellitus, hypertension, and heart failure, and those obese children or adolescents can become obese adults [3–5]. Therefore, it is vital to identify potential risk factors contributing to obesity to reduce the prevalence and mortality rates in obesity-related diseases. Although genetic predisposition, physical activity, and diet play an essential role in the occurrence of obesity, there is still a need for further explanation. More evidence indicates that environmental endocrine-disrupting chemicals might increase the occurrence of obesity [6–9]. Twum et al. demonstrated an underlying relation between exposure to 2,5-dichlorophenol (2,5-DCP) and obesity in children [4]. A significant association was found between bisphenol A (BPA) and general and abdominal obesity [10]. Deierlein showed that phthalates—specifically low-molecular weight phthalates (monoethyl phthalate [MEP], a metabolite of diethyl phthalate (DEP); mono-n-butyl phthalate [MBP], a metabolite of di-n-butyl phthalate (DBP), and mono-isobutyl phthalate [MiBP], a metabolite of di-isobutyl phthalate (DiBP))—had slight associations with girls’ anthropometric outcomes [11]. These substances are readily present in our daily lives, since consumer products usually use parabens as preservatives, building and food packaging materials use phthalates as plasticizers, and the production of pharmaceutical and agricultural products uses 2,5-DCP as a chemical intermediate [12–14]. We can easily contact these environmental endocrine-disrupting chemicals via gastrointestinal intake, dermal contact, and applying products that contain these chemicals [15, 16]. However, most of the previous research studied only a unitary exposure or a set of similar exposures [17–19]. We are exposed to all kinds of chemical exposures simultaneously, which can result in complicated interactions. Therefore, it is necessary to use a suitable statistical model for risk assessment of exposure and obesity [20–22].

We collected data on urinary chemicals or metabolites that had been reported to have an effect on obesity in the National Health and Nutrition Examination Survey (NHANES) from 2005 to 2010. We studied 9 chemical exposures including phenols (BPA, benzophenone-3 (BP-3)), parabens (methylparaben (MeP), propyl paraben (PrP)), pesticides (2,5-DCP, 2,4-DCP) and phthalate metabolites (Mono-benzyl phthalate (MBzP), MEP, MiBP). We selected three statistical methods, including generalized linear regression, weighted quantile sum (WQS) regression, and Bayesian kernel machine regression (BKMR) models, to better analyze multi-exposures’ co-function on adolescent obesity. Among them, BKMR model can resolve the non-linear and complicated interactions between chemical exposures and get more accurate results comparing with the generalized linear regression [23]. All of these three methods have their own advantages and disadvantages, and we expected that this comprehensive analysis would yield insightful and fruitful conclusions.

## Methods

### Study sample

The NHANES is a cross-sectional nationally representative program, aiming to collect information on adults’ and children’s health and nutritional condition in the United States, which is reviewed and approved by the National Center for Health Statistics, as one of the departments of Centers for Disease Control and Prevention (CDC). The NHANES program was conducted in the early 1960s and released the data in biennial datasets. In order to get the unbiased national health information on the non-institutionalized population of the United States, the NHANES used a considerate, multi-stage stratification probability sampling design [24]. We collected publicly accessible data from 2005 and 2010. We selected participants between 6 and 19 years old, with attainable measurements of urinary phenols, parabens, pesticides, and phthalate metabolites Body mass index (BMI) and waistcircumference simultaneously (*n* = 2629) and excluded the participants whose data on covariates, including age, gender, race, education level, family income-to-poverty ratio, caloric intake, serum cotinine, and urinary creatinine, were missing (*n* = 257). Finally, 2372 participants were included in our study.

### Measurement of chemical exposures

Urinary samples were collected and stored at − 20 °C. They were sent to the National Center for Environmental Health, the Organic Analytical Toxicology Branch, for analysis. BPA, BP-3, MeP, PrP, 2,4-DCP, and 2,5-DCP,were extracted by on-line solid-phase extraction (SPE). They were measured by high-performance liquid chromatography as well as tandem mass spectrometry (MS/MS). MBzP, MEP, and MiBP were measured by high-performance liquid chromatography-electrospray ionization-tandem mass spectrometry (HPLC-ESI-MS/MS). The limit of detection (LOD) for the compounds to be analyzed, including BPA, BP-3, MeP, PrP, 2,4-DCP, and 2,5-DCP, were 0.4 ng/mL, 0.4 ng/mL, 1.0 ng/mL, 0.2 ng/mL, 0.2 ng/mL, 0.2 ng/mL in the data from 2005 to 2010, respectively. And the LOD for MBzP, MEP, and MiBP were 0.3 ng/mL, 0.8 ng/mL, and 0.3 ng/mL in the data from 2005 to 2008 and 0.2 ng/mL, 0.4 ng/mL, and 0.2 ng/mL in the data from 2009 to 2010. These values below the limit of detection were divided by the square root of 2 to replace the original values. As one study recommended [25], we treated urinary creatinine as a covariate to explain the urinary dilution. Urinary creatinine was measured by a Beckman Synchron CX3 Clinical Analyzer. The NHANES provides detailed information on the measurement method in the section on laboratory methods on its website [26, 27].

### Anthropometric variables

Trained health technicians measured the body weight and height according to the standardized protocol. The BMI was calculated using each person’s weight in kilograms to divide the square of their height in meters. However, because the standard BMI shows differences for the different ages and gender among children, measuring BMI percentiles and the BMI z-score was more appropriate. The BMI z-score was calculated in regards to the children’s age, gender, and BMI. An appropriate standard was used, which reflected the number of SDs differing from the mean of the BMI with reference to the same age and gender. The methodology to calculate the BMI z-score specifically for different ages and gender was provided by the CDC [28]. We defined a child to be obese when their BMI was above or equal to the 95th percentile for their age and gender in accordance with the CDC recommendations [29].

### Covariates

Covariates, including age, gender, race, education level, family income-to-poverty ratio, caloric intake, serum cotinine, and urinary creatinine, were collected by interview or laboratory detection by NHANES. Race was grouped into Mexican American, Other Hispanic, Non-Hispanic White, Non-Hispanic Black, and Other Race. Education level was categorically grouped into ≤ 5 grade, 6 − 8 grade, 9 − 12 grade, or High School Graduate with No Diploma, High School Graduate and GED or Equivalent, or More than high school. The family income-to-poverty ratio was divided into three groups: ≤ 1.30, 1.31 − 3.50, and > 3.50. The caloric intake was dichotomously divided into normal intake and excessive intake, according to the Dietary Guidelines for Americans 2010 [30]. Serum cotinine indirectly reflected the exposure to environmental tobacco. Serum cotinine, age, and urinary creatinine were considered to be continuous variables.

### Statistical analysis

We used the χ^2^ test and the t-test to analyze categorical variables and continuous variables, respectively. And for the serum cotinine and urinary creatinine, we used wilcoxon rank-sum test. We calculated the descriptive statistics on BPA, BP-3, MeP, PrP, 2,4-DCP, 2,5-DCP, MBzP, MEP, and MiBP. Because the distributions of the chemical exposures were skewed, we log-transformed the concentrations of all chemical exposures. We used the Pearson correlation to calculate the correlation coefficients among all chemical exposures. *p* < 0.05 was considered to be statistically significant.

### Generalized linear regression

We conducted multivariable logistic regression to analyze each chemical exposure and the odds ratios (ORs) of obesity in different quartiles. We also fitted a multivariable linear regression model to assess the association between each chemical exposure and the continuous variable of the BMI z-score in different quantiles. In addition, we fitted the models, adjusting for all the chemical exposures. All the regression models were adjusted by age, gender, race, education level, family income-to-poverty ratio, caloric intake, serum cotinine, and urinary creatinine. We used log-transformed urinary creatinine as an independent covariate instead of the creatinine-adjusted concentration [25].

### Weighted quantile sum (WQS) regression

The WQS model scored all the chemical exposures into quantiles and estimated the weight index:

$$ \mathcal{g}\left(\mu \right)={\beta}_0+{\beta}_1\left(\sum \limits_{i=1}^c{\omega}_i{q}_i\right)+{z}^{\prime}\varphi, $$

where *ℊ*() represents any monotonic link function, μ is the predictable variable, *ω* is the weight of the *i*th components to be estimated, *q*_*i*_ refers to different quantiles, and $\left(\sum \limits_{i=1}^c{\omega}_i{q}_i\right)$ represents the weight quantile sum of the set of *c* components of interest. Furthermore, *β*_1_ denotes the regression coefficient for the weight quantile sum, *β*_0_ is the intercept, *z*^′^ refers to the covariates, including risk factors and confounders, and *φ* is the coefficients for the covariates. The weights were estimated between 0 and 1, and added up to 1. In this study, we divided the data into the training set (40%) and the validation set (60%), we also set *β*_1_ to be positive and the seed was set to be 2019. Besides, we also constrained *β*_1_ to be negative to find if there was a significant relationship in this way. We bootstrapped the training set 10,000 times and got the estimated weights, which maximized the likelihood of the non-linear model. A significant level (*p* < 0.05) was set to test the significance of the weights in each bootstrap. We calculated the ${\overline{\omega}}_i$ to estimate the weight quantile sum:

$$ \mathrm{WQS}=\sum \limits_{i=1}^c{\overline{\omega}}_i{q}_i $$

$$ {\overline{\omega}}_i=\left(1/{n}_B\right)\sum \limits_{j=1}^{n_B}{\omega}_{ij,} $$

where *n*_*B*_ represents the number of bootstraps in which *β*_1_ was significant. The estimated WQS was then determined using the validation set. All the chemical exposures were included in the model, and a specific weight was calculated for each component, representing their contribution to the WQS index. The chemical exposures included were constrained to have the same effect with the outcome (all positive or all negative) [31].

### Bayesian kernel machine regression (BKMR)

The BKMR model utilizes a non-parametric approach to flexibly model the association between chemical exposures and healthy outcomes, including the nonlinear and/or interactions in the exposure-outcome association. A high-dimension exposure-response relationship induced by multiple variables incorporated in the model would make it difficult to ascertain the basis function. Thus, we used a kernel machine regression:

$$ {Y}_i=h\left({z}_i\right)+{x}_i\beta +{\epsilon}_i, $$

where *Y*_*i*_ is the health outcome, *i* refers to the individual (*i* = 1, 2, 3…n), *z*_*i*_ is the chemical exposures, *x*_*i*_ is the potential confounders, and *β* represents the effect of the covariates. *ϵ*_*i*_ is the residual that obeys the normal distribution N (0, *σ*^2^). *h*() is the function that fits the exposure and the outcome considering nonlinear and interactions between the exposures. We grouped the chemical exposures into three groups (group1: BPA, BP-3, MeP, and PrP; group2: 2,5-DCP and 2,4-DCP; group3: MBzP, MEP, and MiBP), according to their source and correlation (chemical exposures with high correlation were grouped) with each other. A hierarchical variable selection approach was used to estimate the posterior inclusion probability of highly correlated variables, which was based on our prior knowledge. The model was fit with 10,000 iterations using a Markov chain Monte Carlo (MCMC) method. The parameter r.jump2 was separately set to 0.2 (in the BMI z-score model) and 0.001 (in the obesity model) to get suitable acceptance rates.

We also analyzed the association between the quantiles of the chemical exposures and binary healthy outcome (obesity and non-obesity) using a probit BKMR model:

$$ {\Phi}^{-1}\left({\mu}_i\right)=h\left({z}_i\right)+{x}_i\beta, $$

where Φ^−1^ is the link function and *μ*_*i*_ is the probability of the binary outcome [22, 23].

Trace plots of parameter in both BMI z-score and obesity model were visualized to investigate the convergence.

All of the statistical analysis were conducted using R software (version 3.6.0).

## Results

There were 2372 children and adolescents included in our study. The general characteristics of the participants are presented in Table 1. The prevalence of obesity was 20.53%. It showed that the mean age of obesity and non-obesity is close: approximately 12-and-a-half years old. About half (44.98%) of the participants were ≤ 5 grade, and 53.03% had a normal caloric intake. The mean (SD) BMI and waist circumferences were 30.41 (6.99) and 96.17 (18.05) cm in the obesity group and 19.68 (3.66) and 69.79 (11.36) cm in the non-obesity group, respectively. The mean (SD) BMI z-scores were 2.12 (0.32) in the obesity group and 0.18 (0.94) in the non-obesity group. There were significant differences between the obesity and non-obesity participants in terms of race, family income, caloric intake, urinary creatinine, BMI, BMI z-score, and waist circumference.

**Table 1:** Demographic characteristics of the NHANES 2005–2010 participants (*N* = 2372), aged 6–19 years

Characteristics	Obesity 487 (20.53%)	No obesity 1885 (79.47%)	*P* value
**Age (y), mean (SD)**	12.57 (3.81)	12.51 (4.01)	0.729
**Gender**			0.931
Male	252 (10.62%)	982 (41.40%)	
Female	235 (9.91%)	903 (38.07%)	
**Race**			< 0.001
Mexican American	143 (6.03%)	516 (21.75%)	
Other Hispanic	52 (2.19%)	152 (6.41%)	
Non-Hispanic White	112 (4.72%)	615 (25.93%)	
Non-Hispanic Black	157 (6.62%)	491 (20.70%)	
Other Race	23 (0.97%)	111 (4.68%)	
**Education level**			0.155
≤5 grade	215 (9.06%)	852 (35.92%)	
6–8 grade	118 (4.97%)	409 (17.24%)	
9–12 grade, No Diploma	115 (4.85%)	453 (19.10%)	
High School Graduate	26 (1.10%)	79 (3.33%)	
GED or Equivalent, More than high school	13 (0.55%)	92 (3.88%)	
**Family income-to-poverty ratio**			0.003
≤ 1.30	235 (9.91%)	774 (32.63%)	
1.31,3.50	177 (7.46%)	706 (29.76%)	
> 3.50	75 (3.16%)	405 (17.07%)	
**Caloric intake**			0.014
Normal intake	283 (11.93%)	975 (41.10%)	
Excessive intake	204 (8.60%)	910 (38.36%)	
**Serum cotinine (ng/mL), GM (SD)**	0.13 (10.55)	0.13 (14.48)	0.140*
**Urinary creatinine (mg/dL), GM (SD)**	119.89 (1.88)	107.53 (2.01)	0.005*
**BMI, mean (SD)**	30.41 (6.99)	19.68 (3.66)	< 0.001
**BMI z-score, mean (SD)**	2.12 (0.32)	0.18 (0.94)	< 0.001
**Waist Circumference (cm), mean (SD)**	96.17 (18.05)	69.79 (11.36)	< 0.001

NHANES: National Health and Nutrition Examination Survey; BMI: body mass index. Data are presented as mean ± SD or Geometric mean ± SD or n (%). The t-test and *χ*^2^ test were between the general obesity and no obesity groups. *Wilcoxon rank-sum test was used for the non-normal distribution data

The LOD and the detection frequency of the chemicals above the LOD are shown in Table 2. The detection frequency of MEP (99.9%) had the highest detection frequency of chemical exposures and the detection frequency of all chemical exposures was above 90%. Table 2 also shows the geometric mean, the mean, and the distribution of the chemical exposures. The highest and the lowest geometric means of the chemical exposures were related to the MEP (87.12) ng/mL and 2,4,5-TCP (0.09) μg ∕ L.

**Table 2:** Distribution of the chemical exposures in NHANES 2005–2010 (*N* = 2372)

Chemical exposures	LOD (ng/mL)	DF (%)	GM	Mean	Min	P5	P25	P50	P75	P95	Max
**Phenols (ng/mL)**											
BPA	0.4	95.7%	2.36	4.27	0.28	0.40	1.28	2.30	4.20	12.99	241.00
BP-3	0.4	99.3%	16.49	272.60	0.28	1.40	4.90	12.40	40.60	543.40	94,100.00
**Paraben (ng/mL)**											
MeP	1.0	99.4%	62.66	278.80	0.71	4.50	17.00	58.10	228.20	1119.00	14,900.00
PrP	0.2	95.5%	7.32	59.44	0.14	0.20	1.40	6.50	38.18	283.45	4150.00
**Pesticides (μg/L)**											
2,5-DCP	0.2	99.1%	16.15	255.10	0.14	0.80	3.50	12.20	54.63	955.45	19,400.00
2,4-DCP	0.2	93.5%	1.38	7.01	0.14	0.14	0.50	1.10	2.80	25.58	1230.00
**Phthalate metabolites (ng/mL)**											
MBzP	0.3^a^	99.7%	13.78	30.19	0.15	1.51	6.54	14.83	31.54	93.26	3806.57
MEP	0.8^a^	99.9%	87.12	252.60	0.37	11.42	33.84	76.73	209.97	1027.72	11,810.04
MiBP	0.3^a^	99.7%	9.98	20.38	0.21	1.50	5.20	10.81	20.31	51.62	6286.00

*NHANES* National Health and Nutrition Examination Survey, *LOD* Limit of detection, *DF* Detection frequency, *GM* Geometric mean

^a^The LOD for MBzP, MEP, and MiBP were 0.3 ng/mL, 0.8 ng/mL, and 0.3 ng/mL in the data from 2005 to 2008 and 0.2 ng/mL, 0.4 ng/mL, and 0.2 ng/mL in the data from 2009 to 2010

**Fig. 1:**
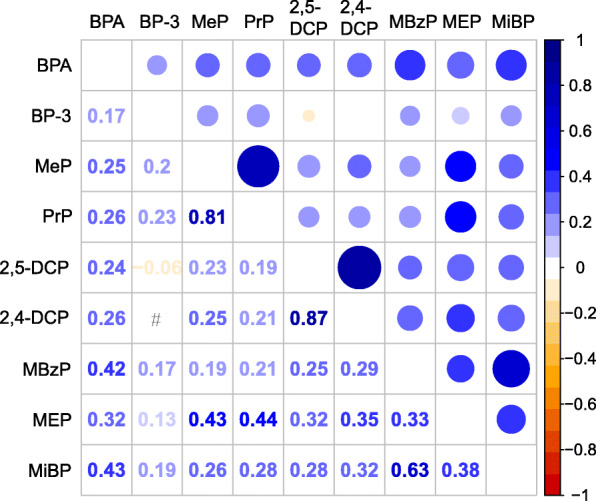
Pearson’s correlations among the urinary concentrations of 9 chemical exposures or metabolites (*N* = 2372), NHANES, USA, 2005–2010. All the correlations were statistically significant (*P* < 0.05), except those of BP-3 and 2,4-DCP (*P* = 0.69). #: *P* > 0.05

We found significant correlations (*P* < 0.05) among 9 chemicals (Fig. 1), except for the correlation between BP-3 and 2,4-DCP (*P* = 0.69). There was a positive correlation between other compounds, except for a nearly no correlation of BP-3 with 2,5-DCP (*r* = − 0.06). 2,5-DCP was found to have a strongly correlation with 2,4-DCP (*r* = 0.87). Additionally, a high correlation between MeP and PrP (*r* = 0.81) was found.

The results from the multivariable logistic and linear regression models adjusted for the covariates are shown in Tables 3 and 4, respectively. The adjusted multivariable logistic regression analysis revealed a statistically significant association between obesity and MeP (OR (95% CI): 0.80 (0.68, 0.94)), 2,5-DCP (OR (95% CI): 1.25 (1.11, 1.40)), MEP (OR (95% CI): 1.28 (1.04, 1.58)), and MiBP (OR (95% CI): 1.42 (1.07, 1.89)), with MeP showing a negative association with dichotomous variable obesity. PrP was found to have a negative association with obesity only when comparing the 4th quartile with the reference quartile (OR (95% CI): 0.69 (0.49, 0.98)). When comparing the 2nd, 3rd, and 4th 2,5-DCP quartiles with the reference quartile, 2,5-DCP had a higher odds ratio (OR (95% CI): 1.49 (1.07, 2.07); 1.80 (1.30, 2.51), and 2.06 (1.47, 2.89), respectively) (Table 3). When comparing the second, third, and fourth quartiles of MEP with the reference quartile, MEP had a higher odds ratio (OR (95% CI): 1.04 (0.75, 1.43); 1.28 (0.92, 1.79), and 1.39 (0.98, 1.98), respectively; Table 3). We used adjusted multivariable linear regression to evaluate the relation between 9 chemical exposures and the BMI z-score (Table 4). We found MeP (second vs. first quartile) to be negatively associated with the BMI z-score (*β* (95% CI): − 0.14 (− 0.27, − 0.01)), and 2,5-DCP (third vs. first quartile) as well as MEP to be positively associated with the BMI z-score (*β* (95% CI): 0.16 (0.02, 0.30); 0.12 (0.02, 0.21), respectively). The second, third, and fourth MEP quartiles had a higher BMI z-score (*β* (95% CI): 0.02 (− 0.12, 0.16); 0.12 (− 0.03, 0.27), and 0.14 (− 0.02, 0.30), respectively) compared with the lowest reference quartile (Table 4).

**Table 3:** Association between single exposure and obesity in the NHANES 2005–2010 (*N* = 2372)

Chemical exposures	Quartile 1	Quartile 2		Quartile 3		Quartile 4		Total	
		OR (95%CI)	*P* value	OR (95%CI)	*P* value	OR (95%CI)	*P* value	OR (95%CI)	*P* value
**Phenols**									
BPA	Ref	0.95 (0.70, 1.30)	0.759	0.92 (0.66, 1.27)	0.595	1.05 (0.75, 1.47)	0.770	1.05 (0.80, 1.38)	0.728
BP-3	Ref	1.00 (0.74, 1.34)	0.984	1.18 (0.87, 1.59)	0.282	0.93 (0.68, 1.28)	0.655	0.98 (0.84, 1.12)	0.738
**Paraben**									
MeP	Ref	0.69 (0.51, 0.92)	0.013	0.65 (0.47, 0.88)	0.006	0.63 (0.45, 0.88)	0.007	0.80 (0.68, 0.94)	0.006
PrP	Ref	1.04 (0.78, 1.40)	0.784	0.82 (0.60, 1.12)	0.218	0.69 (0.49, 0.98)	0.037	0.90 (0.79, 1.03)	0.135
**Pesticides**									
2,5-DCP	Ref	1.49 (1.07, 2.07)	0.017	1.80 (1.30, 2.51)	0.001	2.06 (1.47, 2.89)	0.001	1.25 (1.11, 1.40)	0.001
2,4-DCP	Ref	0.97 (0.70, 1.35)	0.863	1.04 (0.74, 1.45)	0.829	1.11 (0.79, 1.58)	0.536	1.16 (0.97, 1.37)	0.098
**Phthalate metabolites**									
MBzP	Ref	1.07 (0.79, 1.45)	0.683	1.05 (0.76, 1.46)	0.753	0.89 (0.63, 1.27)	0.535	0.96 (0.75, 1.21)	0.705
MEP	Ref	1.04 (0.75, 1.43)	0.824	1.28 (0.92, 1.79)	0.140	1.39 (0.98, 1.98)	0.069	1.28 (1.04, 1.58)	0.022
MiBP	Ref	1.49 (1.08, 2.07)	0.016	1.43 (1.01, 2.03)	0.045	1.62 (1.11, 2.37)	0.013	1.42 (1.07, 1.89)	0.015

*NHANES* National Health and Nutrition Examination Survey, *OR* Odds ratio, *CI* Confidence interval. Total means continuous chemical variable. Multivariable logistic regression was conducted, and odds ratios (ORs) were calculated while comparing the second, third, and fourth quartiles of each chemical with reference to the first exposure quartile (*N* = 2372). Models were adjusted for age, gender, race, educational levels, family income-to-poverty ratio, caloric intake, serum cotinine and log-transformed creatinine

**Table 4:** Association between single exposure and BMI z-score in NHANES 2005–2010 (*N* = 2372)

Chemical exposures	Quartile 1	Quartile 2		Quartile 3		Quartile 4		Total	
		*β* (95%CI)	*P* value	*β* (95%CI)	*P* value	*β* (95%CI)	*P* value	*β* (95%CI)	*P* value
**Phenols**									
BPA	Ref	0.02 (−0.12, 0.16)	0.772	0.01 (−0.14, 0.15)	0.928	−0.01 (−0.15, 0.15)	0.995	−0.06 (−0.19, 0.06)	0.342
BP-3	Ref	0.07 (− 0.07, 0.20)	0.337	0.08 (−0.06, 0.22)	0.259	0.07 (− 0.07, 0.21)	0.325	0.02 (− 0.04, 0.08)	0.541
**Paraben**									
MeP	Ref	−0.14 (− 0.27, − 0.01)	0.044	− 0.14 (− 0.28, 0.01)	0.060	− 0.14 (− 0.30, 0.02)	0.078	−0.05 (− 0.13, 0.02)	0.155
PrP	Ref	−0.01 (− 0.15, 0.12)	0.829	− 0.06 (− 0.20, 0.08)	0.406	−0.10 (− 0.26, 0.05)	0.189	−0.03 (− 0.09, 0.03)	0.394
**Pesticides**									
2,5-DCP	Ref	0.05 (−0.09, 0.19)	0.465	0.16 (0.02, 0.30)	0.023	0.09 (−0.05, 0.24)	0.214	0.03 (−0.03, 0.08)	0.327
2,4-DCP	Ref	−0.05 (− 0.19, 0.10)	0.514	0.06 (− 0.09, 0.21)	0.401	− 0.05 (− 0.20, 0.11)	0.568	−0.02 (− 0.10, 0.06)	0.611
**Phthalate metabolites**									
MBzP	Ref	0.05 (−0.09, 0.18)	0.519	−0.03 (− 0.18, 0.11)	0.653	− 0.02 (− 0.18, 0.14)	0.826	−0.01 (− 0.12, 0.10)	0.862
MEP	Ref	0.02 (−0.12, 0.16)	0.773	0.12 (−0.03, 0.27)	0.108	0.14 (−0.02, 0.30)	0.083	0.12 (0.02, 0.21)	0.017
MiBP	Ref	0.14 (−0.01, 0.28)	0.054	0.07 (−0.08, 0.22)	0.376	0.10 (−0.07, 0.27)	0.251	0.05 (−0.08, 0.18)	0.471

*NHANES* National Health and Nutrition Examination Survey, *CI* Confidence interval; Total means continuous chemical variable. Multivariable linear regression was conducted and regression coefficients (*β*) were calculated while comparing the second, third and fourth quartiles of each chemical with reference to the first exposure quartile (*N* = 2372). Models were adjusted for age, gender, race, educational levels, family income-to-poverty ratio, caloric intake, serum cotinine, and log-transformed creatinine

In the multivariable logistic and linear regression models, including all the chemical exposures, adjusting for the confounding effects of other chemicals, 2,5-DCP, 2,4-DCP, and MEP were found to have a significant association with both the dichotomous variable obesity (OR (95% CI): 1.73 (1.35, 2.24), 0.57 (0.40, 0.82), and 1.35 (1.08, 1.69), respectively) and continuous variate BMI z-score (*β* (95% CI): 0.14 (0.04, 0.24), − 0.20 (− 0.36, − 0.05), and 0.15 (0.05, 0.25), respectively) (see Additional File 1, Tables S1 and S2). We calculated the variance inflation factors (VIFs) (see Additional File 1, Tables S3), and none of them was higher than 10.

**Fig. 2:**
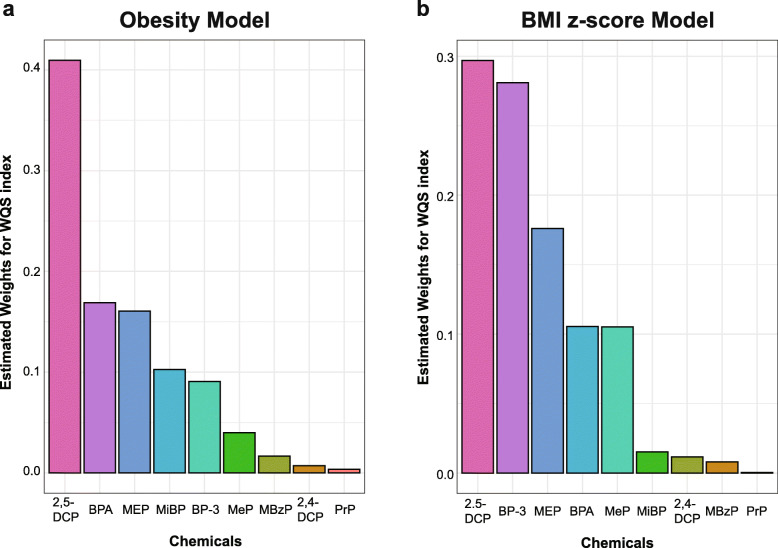
WQS model regression index weights for the obesity (**a**) and BMI z-score (**b**). Models were adjusted for age, gender, race, education levels, family income-to-poverty ratio, caloric intake, serum cotinine, and log-transformed creatinine

We fitted the WQS regression model to the data to evaluate the relationship between the chemical exposures and the outcome in three models, adjusting for different covariates respectively (Table 5). The WQS index had a significant association with obesity in Model 1 (OR (95% CI): 1.50 (1.19, 1.90)). In Model2, the WQS index had a significant association with obesity (OR (95% CI): 1.51 (1.19, 1.91)). In Model 3, the WQS index also had a significantly positive association with obesity after being adjusted for all covariates (OR (95% CI): 1.48 (1.16, 1.89)). We also calculated the estimated chemical weights of the dichotomous variable obesity in obesity model, which are presented in Fig. 2a. The highest weighted chemical in the fully adjusted obesity model was 2,5-DCP (weighted 0.41), followed by BPA and MEP (weighted 0.17 and 0.16, respectively). We also treated the BMI z-score as a continuous variable and fitted the BMI z-score model (Table 5). However, we did not find any significant association between the exposures and the BMI z-score in all three models. The estimated chemical weights of BMI z-score are presented in Fig. 2b. The highest weighted chemical in the BMI z-score model was 2,5-DCP (weighted 0.30). Next to this were BP-3 and MEP, weighted 0.28 and 0.18, respectively. In addition, we also fitted WQS model including all covariates with *β*_1_ constrained to be negative. However, no statistical difference was found in this way. (see Additional File 1, Tables S4).

**Table 5:** Association between the WQS index and obesity in NHANES 2005–2010 (*N* = 2372)

Outcomes	OR/ *β*	95% CI of OR	*P* value
Obesity			
Model 1	1.50	(1.19, 1.90)	< 0.001
Model 2	1.51	(1.19, 1.91)	< 0.001
Model 3	1.48	(1.16, 1.89)	0.002
BMI z-score			
Model 1	0.028	(−0.09, 0.15)	0.643
Model 2	0.033	(−0.09, 0.15)	0.584
Model 3	0.001	(−0.12, 0.12)	0.983

*NHANES* National Health and Nutrition Examination Survey, *CI* Confidence interval. The weighted quantile sum (WQS) regression was fitted for the obesity and BMI z-score, which scored all the chemical exposures into quantiles and estimated the weight index. OR estimates represent the odds ratios of obesity as 1 quartile increased in the WQS index. *β* estimates represent the mean differences in the BMI z-score as 1 quartile increased in the WQS index. Model 1: Adjusted for age, gender, ethnicity, and log-transformed creatinine. Model 2: Adjusted for age, gender, ethnicity, caloric intake, serum cotinine, and log-transformed creatinine. Model 3: Adjusted for age, gender, ethnicity, educational levels, family income-to-poverty ratio, caloric intake, serum cotinine, and log-transformed creatinine

**Fig. 3:**
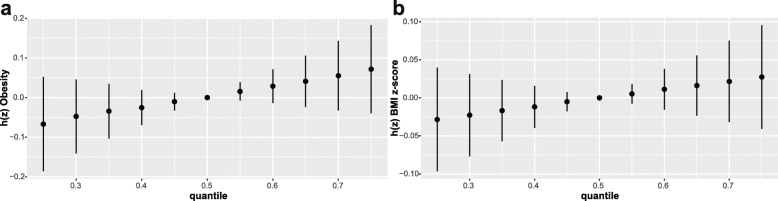
Overall risk (95% CI) of chemical exposures on obesity (**a**) and BMI z-score (**b**) when comparing all the chemicals at different percentiles with their median level. Models were adjusted for age, gender, race, educational levels, family income-to poverty ratio, caloric intake, serum cotinine, and log-transformed creatinine

**Fig. 4:**
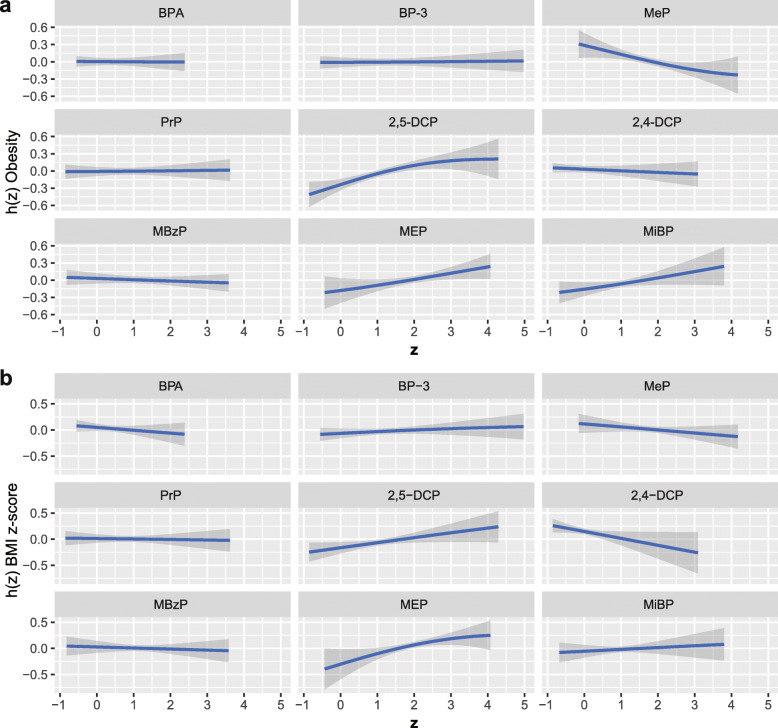
Association and 95% credible intervals for each chemical exposure with obesity (**a**) and BMI z-score (**b**) while fixing other chemical exposures at their median level. The model was adjusted for age, gender, race, educational levels, family income-to-poverty ratio, caloric intake, serum cotinine, and log-transformed creatinine

**Fig. 5:**
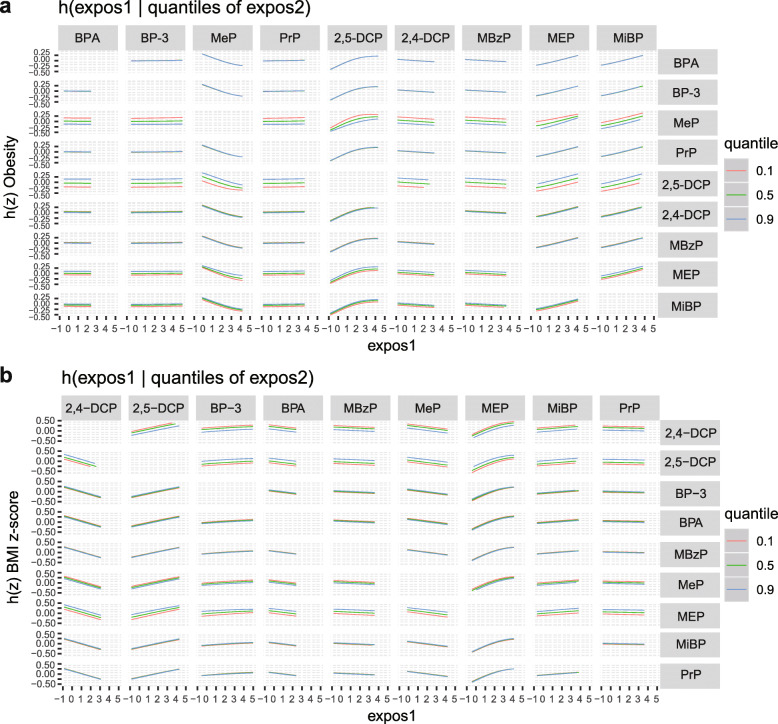
Association between exposure 1 with obesity (**a**) and BMI z-score (**b**), while fixing exposure 2 at the 10th, 50th, and 90th quantiles (and holding the remnant predictors to their median level). The models were adjusted for age, gender, race, educational levels, family income-to-poverty ratio, caloric intake, serum cotinine, and log-transformed creatinine

We grouped 9 chemical exposures into three groups, according to their source and correlation with each other, and fitted the BKMR model to analyze the simultaneous exposure with obesity and BMI z-score. In the obesity model, the group posterior inclusion probabilities (PIP) of the pesticides group was 0.966, while the group PIP of phenol and phthalates metabolites was higher than 0.5 (Table 6). In the pesticides group, 2,5-DCP seemed to drive the effect of the whole group (CondPIP = 0.978; Table 6). In the phthalate metabolites group, MEP drove the main effect of the whole group (CondPIP: 0.656), while MeP drove the main effect in the phenols group (CondPIP = 0.903) (Table 6). The overall association between the chemical mixtures and the binomial outcome is shown in Fig. 3a. We found a positive tendency between chemical exposures and the outcome, in spite of no statistically significant difference. Figure 4 a illustrates the positive associations of 2,5-DCP, MEP, and MiBP with obesity in the BKMR models, while controlling all other chemical exposures at their median level. MeP demonstrated an inverse association with obesity, while no other chemical exposures showed a noteworthy change in slope. We also investigated the relationship between the outcome and a unitary predictor in exposures while fixing another predictor in exposures at the 10th, 50th, and 90th quantiles (and holding the remnant predictors to their median level), and the results are shown in Fig. 5 a. Since the slopes were different between 2,5-DCP and obesity, MEP and obesity while fixing MeP at the 10th, 50th, and 90th quantiles, potential interactions might exist between 2,5-DCP and MeP as well as MEP and MeP. In the BMI z-score model, the values of the group PIP in three groups were 0.329, 0.256, and 0.707, respectively. (Table 6). MEP drove the main effect in its group (CondPIP: 0.831). The overall risk of the chemical mixtures on the outcome are presented in Fig. 3b. Although no statistically significant difference was found, they revealed a positive association of the mixed exposures with the BMI z-score, when we compared all the predictors fixed at different levels with their 50th percentiles. 2,5-DCP and MEP had a trend of a positive association with the BMI z-score, while 2,4-DCP had an inverse association (Fig. 4 b). No obvious interaction was found in the BMI z-score model (Fig. 5 b).

**Table 6:** GroupPIP and condPIP in BKMR model in NHANES 2005–2010 (*N* = 2372)

Chemicals	Group	Obesity		BMI z-score	
		groupPIP	condPIP	groupPIP	condPIP
**Phenols**					
BPA	1	0.775	0.020	0.329	0.278
BP-3	1	0.775	0.046	0.329	0.233
**Paraben**					
MeP	1	0.775	0.903	0.329	0.322
PrP	1	0.775	0.031	0.329	0.166
**Pesticides**					
2,5-DCP	2	0.966	0.978	0.256	0.500
2,4-DCP	2	0.966	0.022	0.256	0.500
**Phthalate metabolites**					
MBzP	3	0.769	0.016	0.707	0.066
MEP	3	0.769	0.656	0.707	0.831
MiBP	3	0.769	0.328	0.707	0.103

*GroupPIP* Group posterior inclusion probability, *condPIP* Conditional posterior inclusion probability, *NHANES* National Health and Nutrition Examination Survey. The three groups in BKMR model were Phenols and paraben (group1), pesticides (group2), and phthalate metabolites (group3). Models were adjusted for age, gender, race, educational levels, family income-to-poverty ratio, caloric intake, serum cotinine, and log-transformed creatinine

To ensure the convergence, we plotted the trace plots, which showed a more or less homogeneously covered space and indicated our model had a good convergence. (see Additional File 1, Fig. 1 and Fig. 2).

For 2,5-DCP and MEP seemed to drive the whole effect in pesticides group (in obesity model) and in phthalate group (in BMI z-score model), we further modeled 2,5-DCP and other groups (phenols group, parabens group, and phthalate group) in obesity model and MEP and other groups (phenols group, parabens group, and pesticides group) in BMI z-score model. The credibility intervals tighten a little (see Additional File 1, Fig. 3 a and b), which meant 2,4-DCP, MiBP and MBzP showed little relevance for the outcome.

## Discussion

Due to the interactions between chemicals, it would be inaccurate to fit only the generalized linear regression model. Therefore, we further used the WQS and BKMR models, which can deal with the interaction between chemicals.

The generalized linear regression showed a positive association between 2,5-DCP, MEP, and MiBP and obesity; however, MeP was negative with the outcome. 2,5-DCP and MEP were significantly associated with the BMI z-score. In the WQS model, 2,5-DCP, BPA, and MEP were found to have relatively high weights in the obesity model, while 2,5-DCP and MEP were found to weight relatively high in the BMI z-score model. In the BKMR model, although no significant association was found between the overall risk of the mixed chemicals and obesity (either obesity or the BMI z-score), there was an upward trend. 2,5-DCP, MEP, and MiBP were found to have a positive association in the obesity model, when fixing others at their median concentration, while in the BMI z-score model, 2,5-DCP, and MEP were positively correlated with the BMI z-score. These results point out the necessity for combining three different models, considering their various advantages and disadvantages.

The generalized linear model, which is used frequently to deal with the exposure-response model, has a fast modeling speed and allowed us to obtain an understandable interpretation of the coefficients. Usually, in the analysis to evaluate the association between exposures and outcome, a unitary exposure or a set of similar exposures is included [12, 32, 33]. Our study included 9 chemical exposures of different sorts. It should be noted that the generalized linear model could not analyze the interactions between exposures. The results may be confusing due to the co-linear or interactions between the exposures.

The WQS mode can include mixed chemicals exposures, with possible high correlations and interactions that are common in real life. In our analysis, 2,5-DCP and MEP were weighted highly in the WQS model. Among these, it is worth noting that BPA and BP-3 were found to weigh highly in the WQS model, yet was found to have a negligible relationship with obesity in the other two models, which may be due to the limitation of the WQS model. The WQS model may lose the full exposure information of the chemical exposures using the quantiles to score the exposures. MeP weighed slightly in the WQS model, which differed from the results in the the other two models. This may result from its negative correlation with the outcome. Since one limitation of WQS is that all chemical exposures included in the model must have the same effective trend with the outcome, otherwise they will be distributed to a negligible weight in the WQS model [34]. In addition, the WQS model may result in a slight weight if a large number of exposures were included, or if there were complex interactions within mixed exposures. Two likely important exposures would have smaller weights if one of them was highly correlated with another one that was assigned a slight weight [31]. However, as for the interactions between chemical exposures, the WQS model still has a high specificity and sensitivity when dealing with mixed predictors, considering the correlated high-dimensional mixtures.

The BKMR model is a new approach to deal with the complexity of mixed exposures, which can analyze not only the exposure-response function of the overall risk of mixed chemical exposures but also the interaction between two chemical exposures. In our study, 2,5-DCP and MEP have a positive association with the continuous variable BMI z-score, which was consistent with the results of our findings in the other two models. However, with the non-linear exposure-response function, other exposures were slightly or negatively associated with the outcomes, which showed consistency with its slight weight in the WQS model. Among the three groups, the MeP was found to have an inverse association with obesity, which is consistent with a previous study [12]. Previous studies could not reach consensus concerning phthalate and BPA, [35–37], and further studies are needed. It is worth noting that MiBP had a positive relationship with the dichotomous variable of obesity but had no relationship with the continuous variable. This may be due to the misleading information when we artificially classified the continuous variable into a dichotomous variable. Besides, we also found potential interactions between 2,5-DCP and MeP as well as MEP and MeP in obesity model, while in the BMI z-score model there was no oblivious interactions. And further investigation is needed on these interactions. The BKMR model also has some limitations. An inconspicuous overall risk association may be observed when exposures which were positive with the outcome or were negative with the outcome both exist [22].

There were several limitations to our study. First, because of the design of the cross-sectional survey project, which collected all of the data at a single time point, there was a limit to the inference of the causation between the chemical exposures and obesity. Second, we used the education level of the individuals themselves instead of their parents’ education level, which can be a factor, since parental education can change their intention to alter the obesity risk factor [38]. Third, chemical concentrations below the limit of detection were simply replaced by the value of the limit of detection divided by the square root of 2, which may cause inaccurate results. Thus, we selected chemical exposures with a high detection frequency. Fourth, obesity is the result of a combination of the long-term effects of various factors. We determined that the concentration of various exposures in urine does not justify a full inference about the mixed chemical exposures on individuals. Further prospective studies are required to investigate the long-term exposure.

## Conclusion

Our study uses three statistical models to analyze the mixed chemical exposures with obesity. 2,5-DCP and MEP were found to have a significant association with the outcome in all models, these results may lead to a false conclusion if only one model is considered. Since all of the models have their own advantages and disadvantages, our study confirms the necessity of combining different statistical models when dealing with the effects of mixed exposures on obesity.

## Supplementary information

**Additional file 1 Table S1.** Association between chemical exposures and obesity with all the chemicals included in NHANES 2005–2010 (*N* = 2372). **Table S2.** Association between chemical exposures and BMI z-score with all of the chemicals included in NHANES 2005–2010 (*N* = 2372). **Table S3.** Variance inflation factors (VIFs) in the multivariable logistic and linear regression models, including all the chemical exposures, adjusting for the confounding effects of other chemicals in NHANES 2005–2010 (*N* = 2372). **Table S4.** Association between the WQS index and obesity in negative direction. Figure 1 The change of beta1 parameter values as the sampler runs in BMI z-score model. Figure 2 The change of beta1 parameter values as the sampler runs in obesity model. Figure 3 Overall risk (95% CI) of chemical exposures on obesity (A) and BMI z-score (B) when comparing all the chemicals at different percentiles with their median level.

**Additional file 2.** Datasets generated and analyzed during the current study.

## Data Availability

The dataset supporting the conclusions of this article is included within the article (Additional File 2).

## Abbreviations

2,4-DCP: 2,4-Dichlorophenol
2,5-DCP: 2,5-Dichlorophenol
BP-3: Benzophenone-3
BKMR: Bayesian kernel machine regression
BMI: Body Mass Index
BPA: Bisphenol A
CDC: Centers for Disease Control and Prevention
CI: Confidence interval
DBP: Di-n-butyl phthalate
DF: Detection frequency
DiBP: Di-isobutyl phthalate
GM: Geometric mean
HPLC-ESI-MS/MS: High-performance liquid chromatography-electrospray ionization-tandem mass spectrometry
LOD: Limit of detection
MBP: Mono-n-butyl phthalate
MCMC: Markov chain Monte Carlo
MeP: Methyl paraben
MEP: Monoethyl phthalate
MiBP: Mono-isobutyl phthalate
MS: Mass spectrometry
NHANES: National Health and Nutrition Examination Survey
ORs: Odds ratios
PIP: Posterior inclusion probabilities
PrP: Propyl paraben
SD: Standard Deviation
SPE: Solid phase extraction
VIFs: Variance inflation factors
WQS: Weighted quantile sum
