# Supplementary material for: Using three statistical methods to analyze the association between exposure to 9 compounds and obesity in children and adolescents: NHANES 2005-2010
Source: Environ Health. 2020 Aug 31;19:94. doi: 10.1186/s12940-020-00642-6 (PMC7457345; doi:10.1186/s12940-020-00642-6)
Supplement: Supplementary file 1 — Additional file 1 Table S1. Association between chemical exposures and obesity with all the chemicals included in NHANES 2005–2010 (N = 2372). Table S2. Association between chemical exposures and BMI z-score with all of the chemicals included in NHANES 2005–2010 (N = 2372). Table S3. Variance inflation factors (VIFs) in the multivariable logistic and linear regression models, including all the chemical exposures, adjusting for the confounding effects of other chemicals in NHANES 2005–2010 (N = 2372). Table S4. Association between the WQS index and obesity in negative direction. Figure 1 The change of beta1 parameter values as the sampler runs in BMI z-score model. Figure 2 The change of beta1 parameter values as the sampler runs in obesity model. Figure 3 Overall risk (95% CI) of chemical exposures on obesity (A) and BMI z-score (B) when comparing all the chemicals at different percentiles with their median level. [file 12940_2020_642_MOESM1_ESM.docx]

**Supplementary materials**

**Table S1** Association between exposures and obesity including all exposures in NHANES 2005-2010 (N = 2372).

| **Chemical exposures** | Quartile 1 | Quartile 2 | | Quartile 3 | | Quartile 4 | | Total | |
| --- | --- | --- | --- | --- | --- | --- | --- | --- | --- |
|  |  | OR (95%CI) | P value | OR (95%CI) | P value | OR (95%CI) | P value | OR (95%CI) | P value |
| **Phenols** |  |  |  |  |  |  |  |  |  |
| BPA | Ref | 0.90 (0.65, 1.24) | 0.514 | 0.86 (0.61, 1.21) | 0.397 | 0.96 (0.68, 1.36) | 0.818 | 0.99 (0.75, 1.32) | 0.973 |
| BP-3 | Ref | 0.99 (0.73, 1.34) | 0.943 | 1.24 (0.92, 1.69) | 0.164 | 1.01 (0.72, 1.41) | 0.959 | 1.03 (0.89, 1.19) | 0.708 |
| **Paraben** |  |  |  |  |  |  |  |  |  |
| MeP | Ref | 0.62 (0.44, 0.87) | 0.006 | 0.64 (0.44, 0.94) | 0.024 | 0.70 (0.44, 1.09) | 0.118 | 0.75 (0.59, 0.95) | 0.018 |
| PrP | Ref | 1.21 (0.87, 1.68) | 0.262 | 1.01 (0.68, 1.51) | 0.948 | 0.78 (0.49, 1.25) | 0.297 | 1.03 (0.84, 1.26) | 0.783 |
| **Pesticides** |  |  |  |  |  |  |  |  |  |
| 2,5-DCP | Ref | 1.62 (1.15, 2.30) | 0.007 | 2.26 (1.54, 3.37) | <0.001 | 3.23 (1.99, 5.30) | <0.001 | 1.73 (1.35, 2.24) | <0.001 |
| 2,4-DCP | Ref | 0.73 (0.51, 1.05) | 0.090 | 0.61 (0.40, 0.92) | 0.019 | 0.48 (0.28, 0.80) | 0.005 | 0.57 (0.40, 0.82) | 0.003 |
| **Phthalate metabolites** |  |  |  |  |  |  |  |  |  |
| MBzP | Ref | 0.94 (0.67, 1.30) | 0.688 | 0.88 (0.61, 1.25) | 0.472 | 0.70 (0.47, 1.04) | 0.076 | 0.78 (0.59, 1.01) | 0.062 |
| MEP | Ref | 1.03 (0.74, 1.43) | 0.853 | 1.33 (0.95, 1.88) | 0.100 | 1.55 (1.07, 2.26) | 0.021 | 1.35 (1.08, 1.69) | 0.008 |
| MiBP | Ref | 1.50 (1.07, 2.12) | 0.020 | 1.51 (1.03, 2.23) | 0.034 | 1.77 (1.16, 2.70) | 0.008 | 1.50 (1.10, 2.05) | 0.011 |

NHANES：National Health and Nutrition Examination Survey; OR: odds ratio; CI: confidence interval. Total means continuous chemical variable. Multivariable logistic regression further adjusting other chemicals was conducted and odds ratios (ORs) were calculated while comparing the 2nd, 3rd and 4th quartiles of each chemicals with reference to the first exposure quartile (N =2372). Models were adjusted for age, gender, race, educational levels, family income–to-poverty ratio, caloric intake, serum cotinine and log-transformed creatinine.

**Table S2** Association between exposures and BMI z-score including all exposures in NHANES 2005-2010 (N = 2372).

| **Chemical exposures** | Quartile 1 | Quartile 2 | | Quartile 3 | | Quartile 4 | | Total | |
| --- | --- | --- | --- | --- | --- | --- | --- | --- | --- |
|  |  | $\beta$ (95%CI) | P value | $\beta$ (95%CI) | P value | $\beta$ (95%CI) | P value | $\beta$ (95%CI) | P value |
| **Phenols** |  |  |  |  |  |  |  |  |  |
| BPA | Ref | 0.01 (-0.13, 0.16) | 0.843 | 0.01 (-0.14, 0.16) | 0.947 | -0.02 (-0.17, 0.14) | 0.843 | -0.08 (-0.21, 0.05) | 0.229 |
| BP-3 | Ref | 0.06 (-0.07, 0.20) | 0.354 | 0.09 (-0.05, 0.23) | 0.186 | 0.09 (-0.06, 0.24) | 0.227 | 0.03 (-0.03, 0.10) | 0.311 |
| **Paraben** |  |  |  |  |  |  |  |  |  |
| MeP | Ref | -0.16 (-0.32, -0.01) | 0.033 | -0.15 (-0.32, 0.03) | 0.098 | -0.13 (-0.34, 0.07) | 0.207 | -0.07 (-0.17, 0.04) | 0.220 |
| PrP | Ref | 0.02 (-0.13, 0.17) | 0.788 | 0.01 (-0.17, 0.18) | 0.969 | -0.08 (-0.28, 0.13) | 0.470 | -0.01 (-0.09, 0.08) | 0.895 |
| **Pesticides** |  |  |  |  |  |  |  |  |  |
| 2,5-DCP | Ref | 0.07 (-0.07, 0.21) | 0.351 | 0.18 (0.02, 0.34) | 0.028 | 0.19 (-0.02, 0.40) | 0.072 | 0.14 (0.04, 0.24) | 0.008 |
| 2,4-DCP | Ref | -0.10 (-0.25, 0.05) | 0.193 | -0.04 (-0.22, 0.13) | 0.624 | -0.19 (-0.41, 0.03) | 0.096 | -0.20 (-0.36, -0.05) | 0.009 |
| **Phthalate metabolites** |  |  |  |  |  |  |  |  |  |
| MBzP | Ref | 0.01 (-0.14, 0.15) | 0.951 | -0.09 (-0.25, 0.07) | 0.274 | -0.08 (-0.26, 0.09) | 0.365 | -0.04 (-0.16, 0.07) | 0.462 |
| MEP | Ref | 0.02 (-0.12, 0.16) | 0.740 | 0.14 (-0.01, 0.29) | 0.066 | 0.19 (0.02, 0.35) | 0.027 | 0.15 (0.05, 0.25) | 0.004 |
| MiBP | Ref | 0.14 (-0.01, 0.29) | 0.059 | 0.09 (-0.07, 0.26) | 0.270 | 0.12 (-0.06, 0.30) | 0.198 | 0.06 (-0.08, 0.20) | 0.413 |

NHANES：National Health and Nutrition Examination Survey; CI: confidence interval. Total means continuous chemical variable. Multivariable linear regression further adjusting other chemicals was conducted and regression coefficients ($\beta$) were calculated while comparing the 2nd, 3rd and 4th quartiles of each chemicals with reference to the first exposure quartile (N = 2372). Models were adjusted for age, gender, race, educational levels, family income–to-poverty ratio, caloric intake, serum cotinine and log-transformed creatinine.

**Table S3** Variance inflation factors (VIFs) in the multivariate logistic and linear regression models.

| **Chemical exposures** | VIFs | |
| --- | --- | --- |
|  | obesity | BMI z-score |
| BPA | 1.64 | 1.63 |
| BP-3 | 1.35 | 1.35 |
| MeP | 3.90 | 3.85 |
| PrP | 4.00 | 3.94 |
| 2,5-DCP | 4.59 | 4.13 |
| 2,4-DCP | 5.02 | 4.52 |
| MBzP | 2.04 | 2.06 |
| MEP | 1.84 | 1.82 |
| MiBP | 2.24 | 2.27 |

VIF: Variance inflation factor; Model included all the chemical exposures, adjusting for the confounding effects of other chemicals in National Health and Nutrition Examination Survey (NHANES) 2005-2010 (N = 2372). Models were adjusted for age, gender, race, educational levels, family income–to-poverty ratio, caloric intake, serum cotinine and log-transformed creatinine.

Table S4 Association between the WQS index and obesity in negative direction.

| Outcomes | OR/$\beta$ | 95% CI of OR | P value |
| --- | --- | --- | --- |
| Obesity |  |  |  |
| Model 3 | 0.81 | (0.65, 1.01) | 0.065 |
| BMI z-score |  |  |  |
| Model 3 | -0.04 | (-0.14, 0.07) | 0.495 |

CI: confidence interval. The weighted quantile sum (WQS) regression with $\beta_{1}$ constrained to be negative was fitted for the obesity and BMI z-score, which scored all the chemical exposures into quantiles and estimated the weight index. OR estimates represent the odds ratios of obesity as 1 quartile increased in the WQS index. $\beta$ estimates represent the mean differences in the BMI z-score as 1 quartile increased in the WQS index. Model 3: Adjusted for age, gender, ethnicity, educational levels, family income-to-poverty ratio, caloric intake, serum cotinine, and log-transformed creatinine.

**Fig. 1**


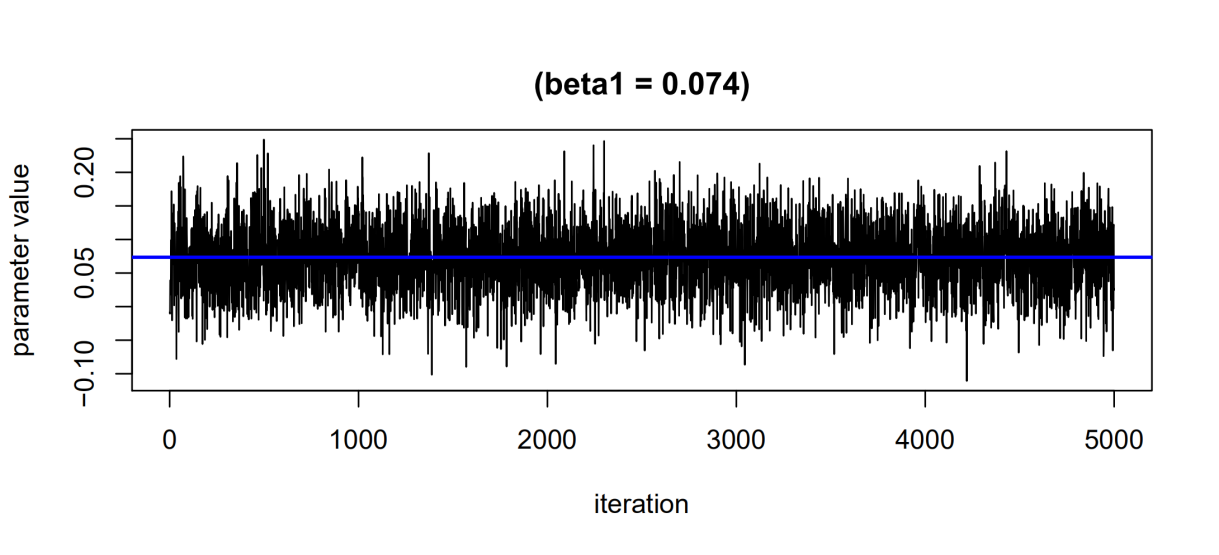


The change of beta1 parameter values as the sampler runs in BMI z-score model.

**Fig. 2**


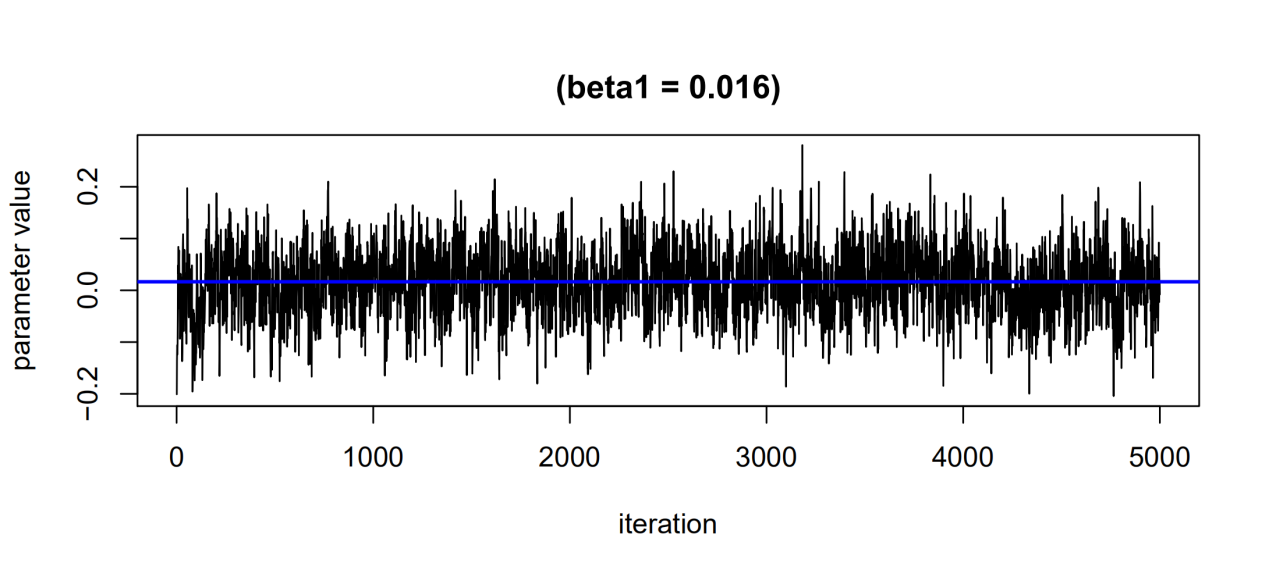


The change of beta1 parameter values as the sampler runs in obesity model.

**Fig. 3**

**
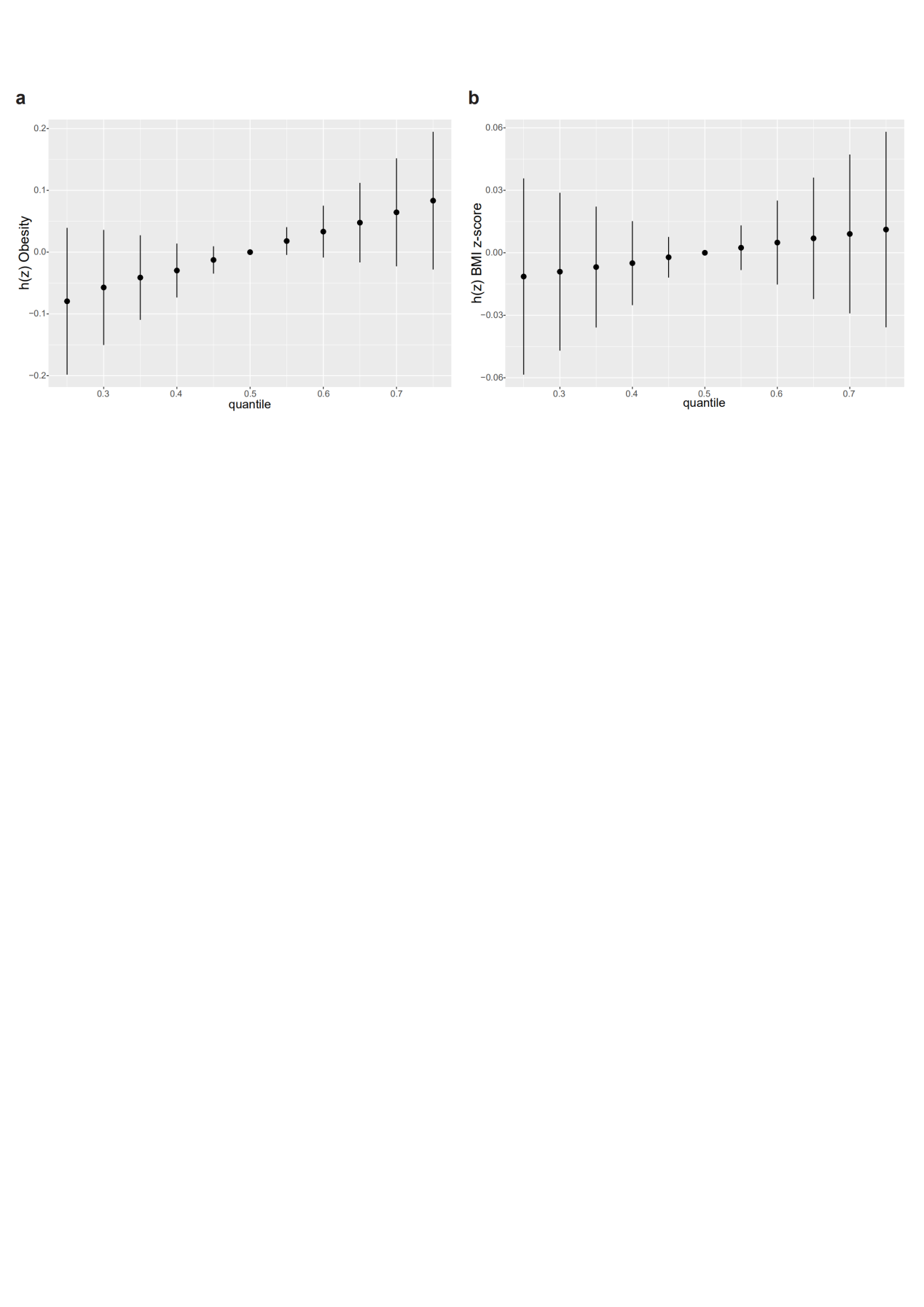
**

Overall risk (95% CI) of chemical exposures on obesity (A) and BMI z-score (B) when comparing all the chemicals at different percentiles with their median level.

2,5-DCP and other groups (phenols group, parabens group, and phthalate group) were included in obesity model. MEP and other groups (phenols group, parabens group, and pesticides group) were included in BMI z-score model.Models were adjusted for age, gender, race, educational levels, family income-to poverty ratio, caloric intake, serum cotinine, and log-transformed creatinine.
